# Skills of general health workers in primary eye care in Kenya, Malawi and Tanzania

**DOI:** 10.1186/1478-4491-12-S1-S2

**Published:** 2014-05-12

**Authors:** Khumbo Kalua, Michael Gichangi, Ernest Barassa, Edson Eliah, Susan Lewallen, Paul Courtright

**Affiliations:** 1 Department of Ophthalmology, University of Malawi, College of Medicine, Blantyre, Malawi; 2 Blantyre Institute for Community Ophthalmology, Lions Sight First Eye Hospital, Blantyre, Malawi; 3 Ministry of Health, Kenya; 4 Kilimanjaro Centre for Community Ophthalmology Tanzania, PO Box 2265, Moshi, Tanzania; 5 Kilimanjaro Centre for Community Ophthalmology International, Division of Ophthalmology, University of Cape Town, South Africa

**Keywords:** primary eye care, cataract, conjunctivitis, presbyopia, trauma, blindness, visual acuity, primary health care worker, health systems, Kenya, Malawi, Tanzania, soins de la vue primaires, cataracte, conjonctivite, presbytie, traumatisme, cécité, acuité visuelle, fournisseurs de soins de santé primaires, systèmes de santé, Kenya, Malawi, Tanzanie

## Abstract

**Background:**

Primary eye care (PEC) in sub-Saharan Africa usually means the diagnosis, treatment, and referral of eye conditions at the most basic level of the health system by primary health care workers (PHCWs), who receive minimal training in eye care as part of their curricula. We undertook this study with the aim to evaluate basic PEC knowledge and ophthalmologic skills of PHCWs, as well as the factors associated with these in selected districts in Kenya, Malawi, and Tanzania.

**Methods:**

A standardized (26 items) questionnaire was administered to PHCWs in all primary health care (PHC) facilities of 2 districts in each country. Demographic information was collected and an examination aimed to measure competency in 5 key areas (recognition and management of advanced cataract, conjunctivitis, presbyopia, and severe trauma plus demonstrated ability to measure visual acuity) was administered.

**Results:**

Three-hundred-forty-three PHCWs were enrolled (100, 107, and 136 in Tanzania, Kenya, and Malawi, respectively). The competency scores of PHCW varied by area, with 55.7%, 61.2%, 31.2%, and 66.1% scoring at the competency level in advanced cataract, conjunctivitis, presbyopia, and trauma, respectively. Only 8.2% could measure visual acuity. Combining all scores, only 9 (2.6%) demonstrated competence in all areas.

**Conclusion:**

The current skills of health workers in PEC are low, with a large per cent below the basic competency level. There is an urgent need to reconsider the expectations of PEC and the content of training.

## Abstract (fr)

**Résumé:**

**Contexte**

En Afrique subsaharienne, les soins de la vue primaires comprennent habituellement le diagnostic, le traitement et l’aiguillage des problèmes oculaires à l’échelon le plus bas du système de santé par des fournisseurs de soins de santé primaires qui reçoivent une formation minimale en soins de la vue dans le cadre de leur formation générale. Nous avons entrepris cette étude dans le but d’évaluer les connaissances en soins de la vue primaires et les compétences en ophtalmologie de ces fournisseurs ainsi que les facteurs y afférents dans des districts donnés du Kenya, du Malawi et de la Tanzanie.

**Méthodes**

Un questionnaire type (couvrant 26 éléments) a été distribué aux fournisseurs de tous les établissements de soins de santé primaires de deux districts de chacun des pays. Des données démographiques ont été recueillies, et un examen visant à mesurer le niveau de compétence des fournisseurs dans cinq tâches clés (reconnaissance et gestion des cas de cataracte avancée, de conjonctivite, de presbytie et de traumatisme grave, et mesure de l’acuité visuelle) a été administré.

**Résultats**

Au total, 343 fournisseurs ont participé à l’étude (100 en Tanzanie, 107 au Kenya et 136 au Malawi). Le pourcentage de fournisseurs atteignant le seuil de compétence requis varie d’une tâche à l’autre : 55,7 % des fournisseurs sont suffisamment compétents pour reconnaître et gérer les cas de cataracte avancée, et ce pourcentage est de 61,2 % pour les cas de conjonctivite, de 31,2 % pour les cas de presbytie et de 66,1 % pour les cas de traumatisme grave. Seuls 8,2 % des fournisseurs peuvent mesurer l’acuité visuelle. Si l’on combine tous les résultats, neuf personnes seulement, soit 2,6 % des fournisseurs, atteignent le seuil de compétence dans les cinq tâches.

Les compétences en soins de la vue primaires actuelles des travailleurs de la santé sont insuffisantes, et une large proportion de ces travailleurs n’atteignent pas le seuil de compétence requis. Il est urgent de réévaluer les attentes en matière de soins de la vue primaires et le contenu de la formation.

## Background

There is a growing body of literature documenting problems in the quality of care in primary health care (PHC) systems in eastern Africa. Murray and Frenk state that most deficiencies in quality of care result from gaps in knowledge or the inappropriate applications of available technology rather than a lack of resources [1]. Gilson and others note serious weaknesses in the quality of PHC in Tanzania [2]. A study there of 502 cases at 62 facilities documented the fact that primary health care workers (PHCW) failed to use standard guidelines in treating about half of severely ill children [3]. With respect to the delivery of eye care, problems have also been documented in the management of urgent eye conditions in PHC facilities [4]. Recently, a pilot study in Tanzania tested knowledge of priority eye conditions among PHCW and found it inadequate to deal with those [5].

In spite of this, there is persistent enthusiasm for the concept of providing eye services (diagnosis, treatment, and referral) at the most basic level of the health system by general PHCW in Africa [6]. PEC may be considered an example of “task shifting” from more specialized workers (dealing only with eye conditions) to less specialized.

The concept of PEC was born after Alma Ata when it was noted that some of the tenets of primary health care could have an impact on reducing two important causes of blindness in developing countries: vitamin A deficiency-related corneal disease and trachoma. Tetracycline eye ointment was included in the basic medicines recommended at the PHC facility to treat the latter. The scope of PEC started to expand when it was noted that general PHCW, with minimal or no equipment, could probably be taught to recognize a white pupil (advanced cataract) and a red eye (which may indicate a number of different problems, some vision threatening and some self-limited) [7]. With the additional skill of measuring visual acuity (VA, a critical indicator of the health of an eye, comparable to vital signs in general medicine) it was assumed that many important eye conditions could be recognized, treated, or referred appropriately at the PHC level. If this were true, it could bring eye care closer to rural patients, and might be expected to contribute significantly to the prevention of blindness and visual impairment efforts. The concept of PEC became very popular with non-governmental organizations and it is frequently cited at meetings as a critical piece of the eye health services in developing countries. The lack of evidence for its benefits as practised, however, has been documented [6].

The curricula for PHCW in many African countries include PEC. The exact content varies, even within countries, and some non-governmental organizations have provided supplementary training. The intent of PEC training is to prepare PHCWs to manage (treat, counsel or refer) anyone who comes to a health centre with an eye complaint. Management of eye conditions by a PHCW is based on history taking, examination of the eye, and knowledge of likely causes. Treatment options usually include antibiotic eye drops or ointment and sometimes steroid drops. Referral options usually include specialised mid-level ophthalmologic personnel (usually ophthalmic clinical officers or ophthalmic nurses) at the district level.

Guided by our pilot study results, we undertook a more comprehensive study that involved more PHCW, extending the evaluation program to another district in Tanzania and to two districts each in Kenya and Malawi. Specifically, we evaluated the knowledge and skills of PHCW to recognize and manage four common eye conditions (cataract, conjunctivitis, presbyopia and trauma) and to perform one skill (measure visual acuity). The four conditions are generally considered to be within the scope of PEC in Africa [8]. Cataract is the major cause of blindness in Africa [9]; conjunctivitis is a common condition which affected 5% in one population-based study in eastern Africa [10]; presbyopia affects around 85% of those over age 50 [11]; and ocular trauma, of unknown incidence, can have serious consequences for vision when treatment is delayed [4].

## Methods

Two districts in each country were selected based on proximity to the lead investigator in the country. All government health centres (known as “dispensaries” in Tanzania) in each district were included and visited by interviewers. On the day of the visit all the PHCW present within these health centres were interviewed. There are sometimes “attendants” and other unofficial cadres of workers at the health centres, and we intended to limit the interviews to officially recognized and trained cadres; this was realistic in Kenya and Tanzania where “unofficial” cadres were infrequent. In Malawi, however, a significant proportion of workers fit this category and we included them since they are regularly providing health care there.

An interview questionnaire for the PHCW was designed and pre-tested in the pilot study [5]. Part 1 of the questionnaire captured demographic information, training history and content, and whether he or she currently manages eye patients. Part 2 was a test of knowledge and skills; 8 points were allotted to testing ability to recognize (1 point) and manage (1 point) four common or important eye conditions (advanced cataract, conjunctivitis, presbyopia and trauma) using large colour photos and a brief history for each. The cataract picture showed an elderly person with white pupil who complained of gradual loss of vision; the conjunctivitis picture showed red “sticky” eyes in someone who complained of itching and watering but no vision loss; presbyopia was a grandmother who looked normal but complained that she could not see well enough to thread a needle or trim her fingernails; the trauma picture showed a large foreign body in the eye of someone with a history of pain and watering after being struck in the eye with a stick. Four more points could be gained from demonstrating how to check visual acuity using a Snellan Chart. (One point was awarded for each of the following: correct distance, measuring each eye separately, recording the visual acuity, and interpreting what it meant). The questions were designed to test a lower threshold for competence, based on the experiences of the authors. Competency in each item required the ability to recognize and manage the condition correctly (2/2 points). For testing visual acuity, competency required performing each of the 4 steps correctly (4/4 points).

The questionnaire was administered by two different interviewers in Tanzania and one each in Malawi and Kenya. The form was translated into Swahili then back into English to ensure accuracy. A Swahili form was used in Tanzania and an English form was used in Malawi and Kenya. The interviewers were trained by the lead investigator in each country, who met together to agree on protocol. A detailed set of instructions for completing the form was prepared.

Data were entered into Microsoft Excel, and then transferred into Stata for analysis. We compared scores among PHCW from each country for each component using chi-square; total scores for health workers were compared among countries by t-test. Mean total scores were used to test associations between competency and other variables, including gender and prior training. Odds ratios for prior training being associated with competency were also calculated.

This study was approved by the Tumaini University ethics committee in Tanzania and by the Ministries of Health in Malawi and Kenya.

## Results

Three-hundred-forty-three PHCW from 137 PHC facilities were studied; these included 59 facilities in Tanzania (100 PHCW), 40 in Kenya (107 PHCW), and 38 in Malawi (136 PHCW). These included all health workers who were present on the day of the interview.

The average number of patients recorded in log books who presented with eye complaints among the centres in 2010 was 52, 108, and 278 in Tanzania, Kenya, and Malawi, respectively. These differences are consistent with the fact that in Tanzania the PHC facility has a catchment population of 2,000-5,000; in Kenya it serves around 10,000, and in Malawi it serves around 8,000-20,000. On average there were 2.7 PHCW per PHC facility (range from 1.9 in Kenya to 3.7 in Malawi). Basic descriptors of the PHCW in each country are provided in Table 1. Of the 312 who said they provide eye care, 110 (35.3%) denied having had PEC training.

**Table 1:** Description of PHCWs

	Tanzania (n=100)	Kenya (n=107)	Malawi (n=136)	All countries (n=343)
Age; mean, SD (%)	45.33 (8.15)	32.75 (8.28)	35.32 (12.2)	37.4 (11.2)

Age; median (range)	46 (29-67)	30 (23-56)	31 (19-77)	35 (23-77)

Sex; # of females (%)	70 (70)	71 (66)	69 (51)	210 (61.2)

Qualification				

Clinical Officer (%)	45 (45)	30 (28)	32 (23)	107 (31.2)

Nurse, MCH worker (%)	55 (55)	77 (72)	38 (27)	170 (49.6)

Attendants (%)	0	0	66 (48)	66 (19.2?

No. who said they had eye training (%)	34 (34)	104 (97)	66 (48)	204 (59.4)

No. who say they provide eye care (%)	100 (100)	106 (99)	106 (78)	312 (91)

No. who mention “test VA” in training (%)	2 (2)	13 (12.1)	13 (9.6)	28 (8.1)

No. who had refresher eye training (%)	5 (5)	3 (2.8)	2 (1.5)	10 (2.9)

Table 2 shows the skills scores for the PHCW. There were significant differences among countries in the scores of PHCW for all components of the score. The percentage of PHCW with “competence” (full points) in each component, presented for Tanzania, Kenya, and Malawi, respectively, was: cataract (66%, 74%, and 34%); presbyopia (35%, 30%, and 29%); conjunctivitis (50%, 84%, and 51%); trauma (68%, 91%, and 46%); and measuring VA (9%, 7%, and 8%). Fewer than 3% of PHCW in each country demonstrated competence in all components together.

**Table 2:** Skill scores for PHCW

	Tanzania N=100	Kenya N=107	Malawi N=136	p-value	All countries (n=343)
Cataract score					

0	22 (22)	25 (23.4)	56 (41.2)		103 (30.0)

1	12 (12)	3 (2.8)	34 (25)		49 (14.3)

2	66 (66)	79 (74)	46 (34)	<0.001	191 (55.7)

Presbyopia					

0	34 (34)	68 (63.5)	56(41.2)		158 (46.1)

1	35 (35)	7 (6.5)	40 (29.4)		82 (23.9)

2	31 (31)	32 (29.9)	40 (29.4)	<0.001	103 (30.0)

Conjunctivitis					

0	28 (28)	11 (10.3)	50 (36.8)		89 (26.0)

1	22 (22)	6 (5.6)	16 (11.8)		44 (12.8)

2	50 (50)	90 (84.1)	70 (51.5)	<0.001	210 (61.2)

Trauma					

0	8 (8)	3 (2.8)	28 (20.6)		39 (11.4)

1	24 (24)	7 (6.5)	46 (33.8)		77 (22.5)

2	68 (68)	97 (90.6)	62 (45.6)	<0.001	227 (66.1)

VA					

0	44 (44)	40 (37.4)	105 (77.2)		189 (55.1)

1	26 (26)	8 (7.5)	8 (5.9)		42 (12.2)

2	12 (12)	27 (25.2)	6 (4.4)		45 (13.1)

3	9 (9)	24 (22.4)	6 (4.4)		39 (11.4)

4	9 (9)	8 (7.5)	11 (8.1)		28 (8.2)

Total score Mean (SD) (12 points possible)	6.36 ( 2.68)	7.34 (2.32)	4.8 (2.96)	<0.001	6.12 (2.21)

Number with 12 points (%)	3 (3)	3 (3)	3 (2)		9 (2.6)

Table 3 shows the association between gender and prior training with total skills scores. In all countries skills scores were higher in males than females. Scores among PHCW who had previous PEC training were higher in all countries but this was only significant in Malawi and when all countries were combined. There was no association between age of PHCW and total skills score (R-squared = 0.0031; p=0.305) in any of the countries.

**Table 3:** Predictors of mean skills score

	Mean score (SD)		Mean score (SD)		Mean score (SD)		Mean score (SD)	
	Tanzania	p	Kenya	p	Malawi	p	All countries	p

Eye training								

Yes	6.71 (2.51)	0.36	7.38 (2.32)	0.20	6.76 (2.44)	<0.001	7.06 (2.40)	<0.001*
							
No	6.18 (2.76)		5.67 (2.08)		2.97 (2.12)		4.55 (2.91)	

Sex								

male	7.60 (2.64)	0.002	8.08 (2.25)	0.02	5.47(3.29)	0.01	6.66 (3.13)	0.002
							
female	5.82 (2.53)		6.95 (2.27)		4.15 (2.44)		5.66(2.67)	

Table 4 shows a significant association between competence and having had PEC training for all components except cataract, where it is reversed; it also shows that 96% of those who have had training are not fully competent.

**Table 4:** Association of PEC training with competence (yes = competence)

	Cataract		Conjunctivitis		Presbyopia		Trauma		Measure VA		All components	
	Yes	No	Yes	No	Yes	No	Yes	No	Yes	No	Yes	No

PEC training	68 (33.3)	136 (66.7)	168 (82.3)	36 (17.6)	70 (34.3)	134 (65.7)	160 (78.4)	44 (21.6)	22 (10.8)	182 (89.2)	9 (4.4)	195 (95.6)

No PEC training	84 (60.4)	55 (39.6)	42 (30.2)	97 (69.8)	33 (23.7)	106 (76.3)	67 (48.2)	72 (51.8)	6 (4.3)	133 (95.7)	0 (0)	139 (100)

OR (95% CI)	0.33 (0.2-0.5)		10.7 (6.5-18)		1.7 (1.0-2.7)		3.9 (2.4-6.2)		2.7 (1.0-6.8)		6.4 (0.8-51.2)	

p-values	<0.001		<0.001		0.04		<0.001		0.03		0.09	

## Discussion

This study is one of few attempting to evaluate the skills in PEC of PHCW in low income countries. PHCW in the study are clinical officers and nurses for the most part, although about half of the PHCW in Malawi had neither of these qualifications. Females comprise at least half and closer to two-thirds in Kenya and Tanzania; PHCW in Tanzania are generally older than in the other countries.

While almost all PHCW said that they provided eye care, not all had been trained to do so. The percentage that received PEC training in Tanzania, Kenya, and Malawi, respectively was 34%, 97%, and 48%. The fact that one-third of those who provide eye treatment have not had any eye training is an example of the overstepping of competence noted by Walter [3]. Most health centres have tetracycline eye ointment and this may provide a false sense of security to PHCW. Unfortunately, many important conditions cannot be treated with tetracycline and such treatment may delay appropriate therapy.

It is interesting that only 28 (41%) of those who were trained in PEC said that testing of VA was part of their training. This is an important skill and often a critical determinant of management of eye conditions; the fact that it was not included in some PEC training requires investigation of the curricula being utilised in each country.

Overall, the scores were low, especially considering that the test was designed only to measure basic competence in common conditions. Presbyopia is likely to be the most common condition in the population (nearly universal in the elderly), but only 30% of PHCW could recognize and manage this. Severe trauma is a condition whose mismanagement can have devastating consequences; it was the condition most readily recognized and managed but even so only 66% of PHCWs were able to do this. Untreated cataract is a major cause of avoidable blindness; the photo showed a white pupil (advanced cataract) but only 56% of PHCW recognized the condition and knew what its management entailed (referral for surgery). Conjunctivitis is a common self-limited condition without visual sequelae and was recognized and managed appropriately by 61%. Ability to measure visual acuity was uniformly low, with fewer than 10% able to demonstrate this critical skill, consistent with the fact that it was not included in PEC training.

Higher scores were significantly associated with having had previous PEC training and with gender (higher scores in males than females). However, the actual differences in mean scores between those with and without training was not large (Table 3) and a large number who had training were still not competent in individual components (Table 4). In recognizing and managing cataract, previous PEC training had a negative effect. There are several possible reasons for the higher competency in males than females. One might be a society gender bias towards health roles: at health centres male workers are viewed more as doctors and as being more confident, while female workers are viewed more as nurses (i.e. assistants, less confident), and this could influence their roles. It is also possible that family responsibilities of the women might cause them to spend less time in the clinic, eventually mastering fewer skills. A similar finding came from a study of PEC workers in Rwanda [12].

There are several limitations to this study. We could not verify the accuracy of the PHCW information on whether they actually had PEC training and what was included in it; some may have said they had no training in the hope that it would be offered and others may have said they did have training since they were providing eye care. We did not attempt to evaluate the quality of training in any of the countries and we believe it to be highly variable. In Malawi, the results must be interpreted in light of the fact that health workers who are not officially part of the system were included; however, they comprise a significant part of the workforce on the ground. It was beyond the scope of this study to determine what constitutes proper training in PEC, but we focused on elements that are generally considered within the scope of PEC in Africa [8].

In spite of the above limitations, these findings raise a number of issues that should be of concern to those who advocate for this form of “task shifting.” First, the training curricula for PEC need to be investigated. Although numerous manuals have been written on PEC, to our knowledge none have been tested in terms of whether they provide the basic skills required to “recognize and manage” eye complaints at the frontline. In a companion paper we provide the first attempt to test the sensitivity and specificity of simple ocular signs and symptoms at identifying eye conditions requiring referral and this area needs more work [13]. A second issue is that PHCW currently appear to be overstepping their competence in treating eye conditions; this is likely made worse by the ready availability of tetracycline eye ointment noted above. Serious consideration needs to be given to what a PHCW can actually be expected to do in terms of providing eye care, considering their limited training, equipment, and numerous other responsibilities.

## Conclusion

In summary, the current skills of PHCW in PEC in Kenya, Malawi, and Tanzania are low, with a large percent below the basic competence level. There are many challenges in training PHCW to provide an acceptable quality of eye care. There is an urgent need to reconsider the concept of PEC before committing more resources to PEC as it is understood in most African countries. This does not mean the concept should be abandoned; rather, we think that the aim of PEC should be to help reduce avoidable blindness and visual impairment. Thus, it must address the major causes of blindness and visual impairment today; there are likely to be skills that PHCW can contribute in this regard. However, additional research is required to better define the role as well as the most effective mechanisms to support this cadre of health care workers.

## List of abbreviations

DFATD: Foreign Affairs, Trade and Development Canada; GHRI: Global Health Research Initiative; IDRC: International Development Research Centre; MCH: Maternal and Child Health; PEC: Primary eye care; PHC: primary health care; PHCWs: primary health care workers; SD: standard deviation; VA: visual acuity

## Competing interests

The authors declare that they have no competing interests.

## Authors' contributions

PC, SL, and EE conceived the research project. All authors contributed to the drafting of the study design and EE, MG, EB, and KK conducted field staff trainings. KK led manuscript writing with assistance from PC and SL. All authors approved the final manuscript.

## Declarations

The publication costs associated with this article are funded by Foreign Affairs, Trade and Development Canada and the International Development Research Centre through the Global Health Research Initiative.

This article has been published as part of *Human Resources for Health* Volume 12 Supplement 1, 2014: Addressing the human resources for health crisis through task-shifting and retention: results from the Africa Health Systems Initiative's research component. The full contents of the supplement are available online at http://www.human-resources-health.com/supplements/12/S1
